# Clinical management of contrast-induced neurotoxicity: a systematic review

**DOI:** 10.1007/s13760-024-02474-4

**Published:** 2024-02-08

**Authors:** Frederick P. Mariajoseph, Jia Xi Chung, Leon T. Lai, Justin Moore, Tony Goldschlager, Ronil V. Chandra, Adrian Praeger, Lee-Anne Slater

**Affiliations:** 1 Department of Neurosurgery, Monash Health, Clayton, VIC Australia; 2 Department of Surgery, School of Clinical Sciences at Monash Health, Monash University, Melbourne, VIC Australia; 3 Monash Imaging, Monash Health, Clayton, Melbourne, Australia; 4 Department of Radiology, School of Clinical Sciences at Monash Health, Monash University, Melbourne, VIC Australia

**Keywords:** Contrast, Neurotoxicity, Encephalopathy, Complication, Adverse event, Endovascular, Treatment, Management

## Abstract

**Background:**

Contrast-induced neurotoxicity (CIN) is an increasingly recognised complication following endovascular procedures utilising contrast. It remains poorly understood with heterogenous clinical management strategies. The aim of this review was to identify commonly employed treatments for CIN to enhance clinical decision making.

**Methods:**

A systematic search of Embase (1947–2022) and Medline (1946–2022) was conducted. Articles describing (i) patients with a clinical diagnosis of CIN, (ii) with radiological exclusion of other pathologies, (iii) detailed report of treatments, and (iv) discharge outcomes, were included. Data relating to demographics, procedure, symptoms, treatment and outcomes were extracted.

**Results:**

A total of 73 patients were included, with a median age of 64 years. The most common procedures were cerebral angiography (42.5%) and coronary angiography (42.5%), and the median volume of contrast administered was 150 ml. The most common symptoms were cortical blindness (38.4%) and reduced consciousness (28.8%), and 84.9% of patients experienced complete resolution at the time of discharge. Management included intravenous fluids to dilute contrast in the cerebrovasculature (54.8%), corticosteroids to reduce blood–brain barrier damage (47.9%), antiseizure (16.4%) and sedative (16.4%) medications. Mannitol (13.7%) was also utilised to reduce cerebral oedema. Intensive care admission was required for 19.2% of patients. No statistically significant differences were observed between treatment and discharge outcomes.

**Conclusions:**

The clinical management of CIN should be considered on a patient-by-patient basis, but may consist of aggressive fluid therapy alongside corticosteroids, as well as other supportive therapy as required. Further examination of CIN management is required to define best practice.

**Supplementary Information:**

The online version contains supplementary material available at 10.1007/s13760-024-02474-4.

## Introduction

With advancements in technology and techniques allowing access to a growing spectrum of pathologies, the rate of endovascular procedures in clinical practice has experienced exponential growth [1, 2]. Contrast-induced neurotoxicity (CIN) is an increasingly recognised complication of procedures requiring iodinated contrast, that presents as a range of neurological symptoms that typically mimics ischaemic stroke, including sensory and motor deficit, aphasia, cortical blindness, and reduced consciousness [3–5].

Contrast-induced neurotoxicity remains a poorly understood clinical entity, with a lack of formalised diagnostic criteria and evidence-base regarding management. A recent survey of clinicians demonstrated that less than 25% were comfortable in treating CIN, and 82.1% agreed that further investigation was required to enhance treatment strategies [6]. In light of this, we conducted this systematic review with the aim of characterising currently employed management strategies, with the ultimate goal of enhancing clinical decision making and patient outcomes.

## Methods

### Ethical approval

This study was conducted in accordance with the Preferred Reporting Items for Systematic Reviews and Meta-Analyses (PRISMA) guidelines [7]. Ethical approval and patient consent were not required for this study.

### Search strategy

A comprehensive literature search of Medline (1946 to December 2022) and Embase (1947 to December 2022) was performed from inception. Key search terms included “contrast”, “neurotoxicity”, “encephalopathy”, “blindness’, and “deficit”, with Boolean operators employed as appropriate. Reference lists of selected papers were also screened to identify additional publications, and duplicate articles were removed.

### Eligibility criteria

Studies were selected for analysis based on the following inclusion criteria: publications reporting (i) patients with a clinical diagnosis of CIN, (ii) with radiological exclusion (CT or MR brain imaging) of other pathologies (most notably ischaemic or haemorrhagic stroke), (iii) sufficient reporting of treatments administered, and (iv) reporting of discharge outcomes. Reports of patients < 18 years of age were excluded from analysis, as well as conference abstracts, case series from which it was not possible to extract individual data, and non-English publications. For the purposes of this review, CIN was defined as the onset of neurological symptoms following iodinated contrast administration, with clinical, biochemical and radiological exclusion of other pathologies, most notably ischaemic stroke. Reports in which it was unclear if CIN was the primary explanation for the symptoms presented (i.e., other differentials were not adequately investigated) were also excluded.

### Screening process

Two investigators (FM and JXC) independently evaluated studies for eligibility according to the eligibility criteria. Titles and abstracts were screened initially. Full text reports were then examined. Where consensus was not able to achieved, a third investigator was consulted. The systematic review platform Covidence (www.covidence.org; Veritas Health Innovation, Melbourne, Australia) was used to facilitate the screening process. Publications that fulfilled eligibility criteria underwent data extraction.

### Data extraction

Data were extracted by two independent investigators (FM and JXC), and were crosschecked. In the event of discrepancy, further discussion and examination was conducted until consensus was reached. An additional investigator was consulted when consensus was not able to be achieved. Extracted data including demographic and procedural variables including age, sex, country of publication, comorbidities, procedure, indication of procedure, contrast type, and contrast volumes were collected. The clinical signs and symptoms of CIN, along with relevant imaging findings, and discharge outcomes were also extracted. Details of clinical management were collected including medications administered, procedures performed and requirement of intensive care admission.

### Outcome measures

The clinical presentation of CIN was separated into individual symptoms. Likewise, management regimens were categorised into individual medications and treatment. Patients requiring mechanical ventilation were assumed to require intensive care support. Patient outcomes were based on symptoms at time of discharge from hospital. Favourable outcome was defined as complete resolution at time of discharge from hospital. Unfavourable outcome was defined as ongoing symptoms at time of discharge or death.

### Quality assessment

Quality assessment of included publications was conducted using a modified version of the *Methodological Quality and Synthesis of Case Series and Case Reports* eight-item questionnaire proposed by Murad et al. [8], which encompasses the domains of selection, ascertainment, causality, and reporting. Reporting items included in the modified tool were the type of contrast administered, time to onset of CIN, radiological exclusion of other pathology, volume of contrast administered, and time course of CIN symptoms. Two independent investigators (FM and JXC) individually assessed all included publications according to the modified tool.

### Statistical analysis

Included cases were pooled and descriptive analysis was performed for patient demographics, comorbidities, procedural details, clinical symptoms of CIN, administered treatments, and discharge outcomes. Administered treatments were assessed for association with length of CIN symptoms and discharge outcomes. Fisher’s exact test and Chi-squared tests were utilised where appropriate to evaluate association between categorical variables. Statistical significance was defined as a *p* value < 0.05. All statistical analyses were performed with Stata/BE (StataCorp LLC, College Station, Texas, USA).

## Results

### Study selection

**Fig. 1:**
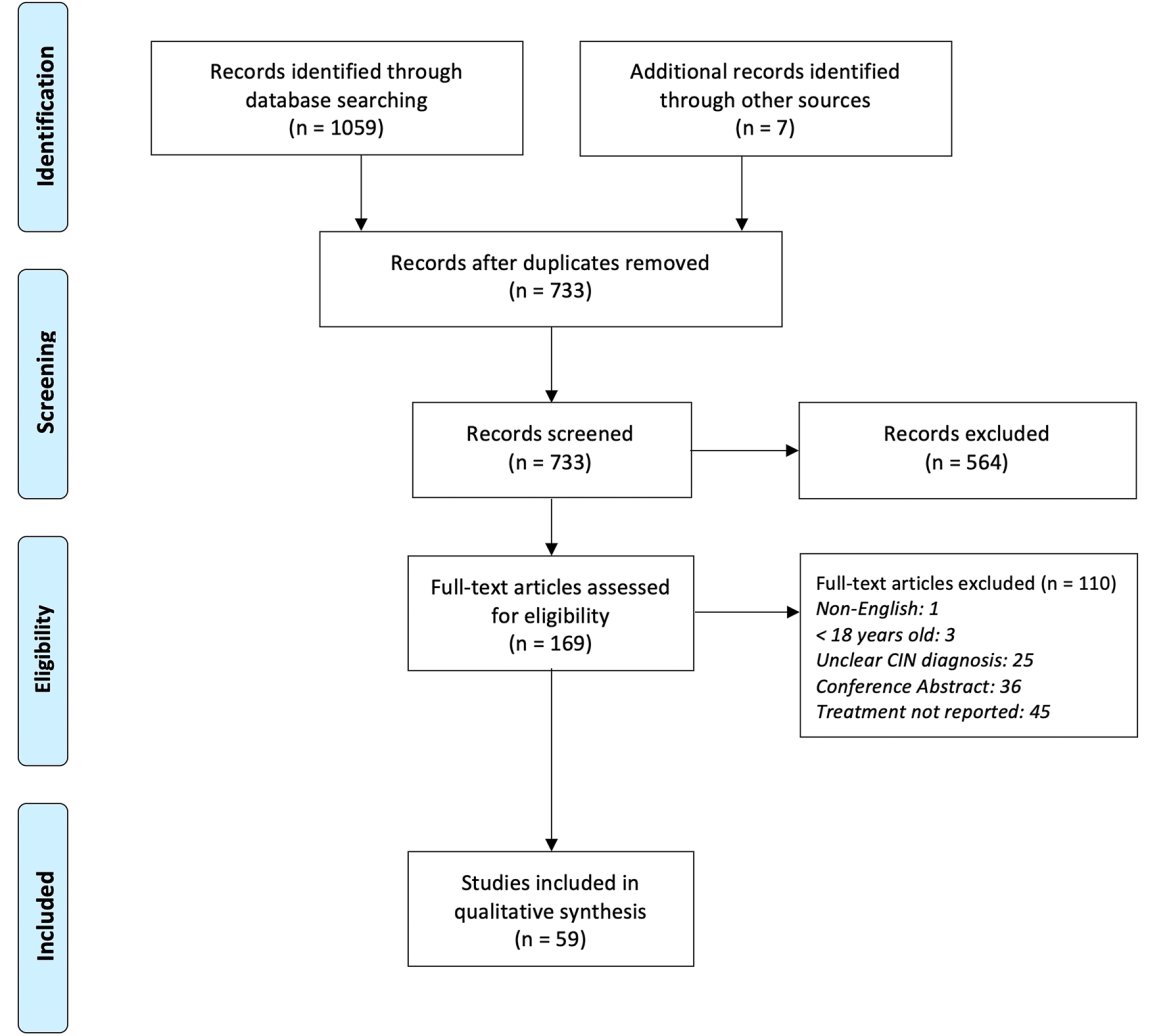
PRISMA flowchart of article search

Our search strategy yielded 1059 articles, and after removal of duplicates, 733 references were screened by title and abstract (Fig. 1). A total of 169 were eligible for full text screening, of which 110 were excluded with reasons. Finally, 59 articles (Supplementary Data 1) were included for final analysis, with a pooled sample size of 73 patients.

### Population characteristics

The median patient age was 64 years (range: 22–89), with 37 (50.7%) males, and 36 (49.3%) females (Table 1). The year of publication ranged from 1995 to 2022, with cases originating from 27 countries. The most commonly reported comorbidities included hypertension (58.9%), diabetes (27.4%), and hyperlipidaemia (24.7%).

**Table 1:** Patient demographics

Variables	*N* = 73
Age, median (range), years	64 (22–89)
Sex	
Male	37 (50.7)
Female	36 (49.3)
Country	
USA	20 (27.4)
Turkey	9 (12.3)
China	7 (9.6)
Japan	6 (8.2)
Italy	4 (5.5)
Australia	2 (2.7)
Belgium	2 (2.7)
India	2 (2.7)
Spain	2 (2.7)
UK	2 (2.7)
Bulgaria	1 (1.4)
Germany	1 (1.4)
Hong Kong	1 (1.4)
Indonesia	1 (1.4)
Ireland	1 (1.4)
Korea	1 (1.4)
Lebanon	1 (1.4)
Malaysia	1 (1.4)
Oman	1 (1.4)
Pakistan	1 (1.4)
Portugal	1 (1.4)
Saudi Arabia	1 (1.4)
Singapore	1 (1.4)
Slovakia	1 (1.4)
Sweden	1 (1.4)
Taiwan	1 (1.4)
Tunisia	1 (1.4)

### Procedural characteristics

Patients underwent a variety of contrast-requiring procedures (Table 2), most notably cerebral angiography with or without intracranial intervention (42.5%), and coronary angiography with or without intervention (42.5%). The most commonly administered contrast agents were iodixanol (20.5%) iopamidol, (17.8%), and iohexol (16.4%). The median volume of contrast administered was 150 ml, with cases reported following as little as 18 ml of contrast.

**Table 2:** Patient comorbidities and procedural details

Variable	*N* = 73
Comorbidities	
Hypertension	43 (58.9)
Diabetes	20 (27.4)
Hyperlipidaemia	18 (24.7)
Chronic kidney disease	11 (15.1)
Prior ischaemic stroke	8 (11)
Hypothyroidism	6 (8.2)
Procedure	
Cerebral DSA ± intervention	31 (42.5)
Coronary angiogram ± intervention	31 (42.5)
Contrast CT	3 (4.1)
Carotid stent	2 (2.7)
Abdominal aortic aneurysm repair	1 (1.4)
Bronchial artery stent	1 (1.4)
Lower limb angiogram	1 (1.4)
Nasopharyngeal tumour embolisation	1 (1.4)
Renal artery angiogram	1 (1.4)
Thoracic aortic aneurysm repair	1 (1.4)
Contrast volume, median (range)	150 (18–1150)
Contrast type	
Iodixanol	15 (20.5)
Iopamidol	13 (17.8)
Iohexol	12 (16.4)
Ioversol	9 (12.3)
Iopromide	8 (11)
Iomeprol	5 (6.8)
Iomeron	1 (1.4)
Iobitridol	1 (1.4)
Iothalamate	1 (1.4)
Not reported	8 (11)

### Clinical course

Commonly reported symptoms of CIN included cortical blindness (38.4%), reduced consciousness (28.8%), hemiparesis (27.4%), confusion (26.0%), and aphasia (23.3%) (Table 3). The median time of symptom onset was 1-h post-procedure, with symptoms appearing as early as intraprocedurally and as late as 27 h following the procedure. At the time of discharge, complete resolution of symptoms was reported in 62 patients (84.9%), nine (12.3%) were discharged with residual deficits, and two patients (2.7%) were deceased. In patients with complete resolution of symptoms, 17 (23.3%) lasted 24 h or less, 25 (34.2%) between 24 and 72 h, and 16 (21.9%) longer than 72 h, with 4 (5.5%) not reporting a timeframe.

**Table 3:** Symptoms and outcomes of CIN

Variable	*N* = 73
Symptoms	
Cortical blindness	28 (38.4)
Reduced consciousness	21 (28.8)
Hemiparesis	20 (27.4)
Confusion	19 (26.0)
Aphasia	17 (23.3)
Agitation	14 (19.2)
Seizure activity	12 (16.4)
Homonymous hemianopsia	4 (5.5)
Inattention/neglect	3 (4.1)
Diplopia	1 (1.4)
Time to Onset of CIN, median (range), hours	1 (intraprocedural – 27)
Discharge outcome	
Resolved in ≤ 24 h	17 (23.3)
Resolved in 24–72 h	25 (34.2)
Resolved in > 72 h	16 (21.9)
Resolved in unspecified timeframe	4 (5.5)
Ongoing symptoms on discharge	9 (12.3)
Died	2 (2.7)

### Management

Several treatment options were reported (Table 4). The most commonly employed treatments included intravenous fluids (54.8%), corticosteroids (47.9%), sedatives (16.4%) and antiseizure medications (ASMs) (16.4%). ASMs were initiated as seizure prophylaxis in 33.3%. Mannitol was utilised in 13.7% of patients, presumably for management of intracranial pressure and cerebral oedema. Seven (9.6%) patients received calcium channel blockers (CCBs). Four patients (5.5%) underwent haemodialysis, all of whom had pre-existing chronic kidney disease. Antipsychotics were administered in 4 patients (5.5%) for management of agitation and confusion. A total of 14 patients (19.2%) required intensive care admission, and 11 (15.1%) required intubation.

**Table 4:** Treatment of CIN

Variable	*N* = 73
Admitted to intensive care unit	14 (19.2)
Intubation/mechanical ventilation	11 (15.1)
Medical therapy	
IV Fluids	40 (54.8)
Corticosteroids	35 (47.9)
Sedative	12 (16.4)
Antiseizure medication	12 (16.4)
Anticoagulation/thrombolysis	11 (15.1)
Mannitol	10 (13.7)
Calcium channel blocker	7 (9.6)
Haemodialysis	4 (5.5)
Antipsychotics	4 (5.5)

The correlation between administered treatments and the clinical outcomes of CIN was assessed. Univariate analysis demonstrated no significant associations between treatments and the outcomes of patients at the time of hospital discharge (Table 5). When limited to a cohort of patients with complete resolution of their symptoms at discharge, administration of mannitol administration was associated with a longer CIN clinical course (*p* = 0.044), with no association detected with other medications (Table 6). The volume of contrast was also not associated with length of symptoms (*p* = 0.774) or overall discharge outcome (*p* > 0.999). Furthermore, a comparison between cardiac and cerebral interventions demonstrated no difference in length of symptoms (*p* = 0.537), or outcomes (*p* = 0.053).

**Table 5:** Association between administered treatments and discharge outcome

Variable	Complete Resolution (*N* = 62)	Death/deficits (*N* = 11)	*p* value
IV fluids	33 (53.2)	7 (63.6)	0.744
Corticosteroids	27 (43.6)	8 (72.7)	0.104
Sedative	9 (14.5)	3 (27.3)	0.374
Antiseizure medication	8 (12.9)	4 (36.3)	0.075
Anticoagulation/thrombolysis	11 (17.7)	0 (0.0)	0.197
Mannitol	7 (11.3)	3 (27.3)	0.168
Calcium channel blocker	5 (8.1)	2 (18.2)	0.283
Haemodialysis	3 (4.8)	1 (9.1)	0.487
Antipsychotics	4 (6.5)	0 (0.0)	> 0.999

**Table 6:** Association between administered treatments and length of CIN symptoms

Variable	≤ 72 h (*N* = 42)	> 72 h (*N* = 16)	*p* value
IV fluids	22 (52.4)	8 (50.0)	> 0.999
Corticosteroids	18 (42.9)	8 (50.0)	0.769
Sedative	6 (14.3)	2 (12.5)	> 0.999
Antiseizure medication	3 (7.1)	3 (18.8)	0.332
Anticoagulation/thrombolysis	6 (14.3)	5 (31.3)	0.156
Mannitol	2 (4.8)	4 (25.0)	0.043
Calcium Channel Blocker	3 (7.1)	2 (12.5)	0.609
Haemodialysis	2 (4.8)	1 (6.3)	> 0.999
Antipsychotics	3 (7.1)	1 (6.3)	> 0.999

### Quality assessment

Of the 59 included publications, 6 (10.2%) reported the selection criteria and specified the incidence at their institution (Table 7). The type of contrast was reported by 89.8% of publications, and the timing of symptom onset was reported in 79.7%. All included publications provided radiological exclusion of other acute intracranial pathologies. The volume of contrast was reported in 89.8% and the outcome and duration of CIN was specified in 86.4% of papers. Only 34 publications (57.6%) were deemed to provide sufficient details to replicate practice.

**Table 7:** Quality assessment

Domain	Leading explanatory questions	Points	*N* = 59
Selection	1. Does the patient(s) represent(s) the whole experience of the investigator (centre) or is the selection method unclear to the extent that other patients with similar presentation may not have been reported?	6	2 (10.2)
Ascertainment	2. Was the exposure adequately ascertained? *Was the type of contrast reported?*	1	53 (89.8)
	3. Was the outcome adequately ascertained? *Was the timing and symptoms of CIN reported?*	1	47 (79.7)
Causality	4. Were other alternative causes that may explain the observation ruled out? *Was radiological evidence presented to rule out other acute pathologies?*	1	59 (100.0)
	5. Was there a challenge/rechallenge phenomenon?	N/A	N/A
	6. Was there a dose–response effect? *Was the volume of administered contrast reported?*	1	53 (89.8)
	7. Was follow-up long enough for outcomes to occur? *Was the duration of CIN symptoms reported?*	1	51 (86.4)
Reporting	8. Is the case(s) described with sufficient details to allow other investigators to replicate the research or to allow practitioners make inferences related to their own practice?	1	34 (57.6)

## Discussion

### Summary of evidence

In this review of 59 articles describing 73 cases of CIN, we found that the mainstays of clinical management consisted of intravenous fluids (54.8%), corticosteroid therapy (47.9%), with other frequently described medications, including mannitol, ASMs and sedatives. One in four patients (19.2%) were admitted into an intensive care unit, and 11 patients (15.1%) required tracheal intubation. Reported treatments had no observed statistical effect on discharge outcomes.

The commonest symptoms of CIN were hemiparesis, cortical blindness, and reduced consciousness, with a median onset of symptoms 1-h post-procedure. Complete resolution of symptoms was reported in 84.9% of cases.

### Pathophysiological mechanisms

The pathophysiology of CIN is unclear, although it has been suggested that the blood–brain barrier (BBB) plays a vital role [9, 10]. Disruption of the BBB allows passage of contrast agents into the central nervous system, allowing it to potentiate neurotoxic effects. One theory for this relates to the oncotic action of hyperosmolar contrast agents leading to shrinkage of endothelial cells and subsequent opening of the tight junctions [11, 12]. Nonetheless, this is unlikely to be the only cause of BBB dysfunction, with CIN observed in patients administered low/iso-osmolar contrast agents. Other factors including increased shear stress caused by hypertension [13], as well as reduced BBB integrity following ischaemic stroke have also been suggested to cause disruption of the BBB [9], allowing passage of contrast agents. In the current review, almost two in three patients were reported to have hypertension, and 10.3% were reported to have suffered a prior ischaemic stroke.

### Principles of management

#### Intravenous fluids

The clinical manifestation of CIN is thought to occur due to the direct effects of contrast on neural cellular function [14]. In addition, several studies have suggested that damage to the BBB secondary to contrast media is directly proportional to the concentration and the length of time the cerebrovasculature is exposed to contrast [15]. As such, reducing the exposure and concentration of contrast agents in cerebral vessels may minimise the effects of contrast on the CNS. Aggressive administration of intravenous fluids may act to dilute and subsequently accelerate the removal contrast agents from the cerebral vascular system. In the current study, intravenous fluids were reportedly used in 55% of patients. Given the widespread use of intravenous fluid therapy in normal clinical practice, it is likely that this number is much higher and was not specified in some case reports.

#### Corticosteroids

Corticosteroids were administered in 47.9% of CIN patients. Glucocorticoids are known to reduce inflammation, and have long been used in inflammatory conditions affecting the CNS, including infective and autoimmune disease processes [16–19]. Corticosteroids have also been demonstrated to increase the integrity of the BBB by enhancing recovery and upregulating synthesis of BBB tight junction proteins [20, 21]. As aforementioned, the BBB appears to play a key role in the pathogenesis of CIN. By reducing damage to the BBB, and potentially preventing the entry of contrast agents, corticosteroids may play an important role in CIN management.

#### Mannitol

In some instances, the signs and symptoms of CIN have reported to be associated with cerebral oedema [22, 23], which is likely due to changes in oncotic pressure following the extravasation of contrast. Following breakdown and passage of contrast agents through the BBB, the relative hyperosmolarity of contrast media will cause a shift between fluid compartments [24]. In the current study, the use of mannitol was seen to be associated with prolonged CIN symptom course (> 72 h). The most likely explanation for this would be that mannitol was utilised in patients with more severe or prolonged CIN. The use of mannitol to lower intracranial pressure and cerebral oedema is already a standard therapeutic option in the management of neurological conditions [25, 26]. The targeted use of mannitol in patients with cerebral oedema secondary to CIN would be expected to improve symptoms associated with the localised effects of cerebral oedema as well as the compression of neural structures secondary to raised intracranial pressure. The findings of this review, ultimately, cannot confirm the benefits of mannitol use in CIN, but may be able to guide management in patients with cerebral oedema.

#### Calcium channel blockers

Calcium channel blockers were reported in approximately 9.6% of cases, and were presumably used to prevent vasospasm. Although the current definition of CIN remains unclear, cerebral vasospasm is a neurological pathology in and of itself, being a major cause of mortality and neurological morbidity [27, 28]. It may be possible that contrast agents induce vasospasm, however, patients with suspected vasospasm following endovascular procedures should be categorised separately to CIN patients, with an arsenal of treatment options and increasingly evidence-based management strategies available to clinicians [27–30]. Further study and understanding of the pathophysiology is required to rationalise the use of CCBs in CIN.

#### Antiseizure medications

The administration of ASMs were primarily used in the management of patients experiencing seizure activity, with two-thirds of patients in this review who received ASM therapy experiencing active seizures as part of their clinical manifestation. The remaining 33.3% were administered ASMs for seizure prophylaxis. Sedating agents, such as benzodiazepines, were also reported in the treatment of seizures associated with CIN. Antiseizure medications are already commonly used prophylactically in neurosurgery and neurotrauma [31–34]. In the current review, seizures were experienced by 16.4% of patients, and forms a recognised part of the clinical picture of CIN. The use of ASMs for seizure prophylaxis in patients with CIN may be warranted, although requires careful consideration of the patient’s clinical state and possible adverse effects.

### Clinical implications

In the clinical management of CIN, the most important step is to exclude other acute intracranial pathologies that require emergent treatment, most notably ischaemic stroke. Once a diagnosis of CIN is clear, several supportive and therapeutic options may be available. Corticosteroids to reduce inflammation and reduce BBB damage, as well as aggressive intravenous fluids to dilute and remove contrast from the cerebrovascular are likely to form the foundation of CIN management moving forward. Depending on the clinical scenario, mannitol may also be appropriate to reduce cerebral oedema. Other medications including ASMs for seizure prophylaxis should also be considered. In patients with high clinical concern or reduced consciousness, admission into an intensive care unit for supportive care and close observation would be appropriate.

In clinical practice, it is vital to individualise treatment. This principle is all the more relevant in the context of CIN, given the relative paucity of literature, and the lack of definitive evidence for treatment strategies. As a result, our recommendation would be to evaluate the clinical manifestation of CIN in each patient and decide on therapy on a case-by-case basis. The literature presented may act as a guide to enhance decision-making, but ultimately, each patient should be treated according to their symptoms and clinical state, until further evidence emerges.

### Study strengths

This review has several strengths. We applied a very strict eligibility criteria to ensure that all included cases of CIN were as accurate as possible, to increase the certainty from which conclusions could be drawn. Patients with insufficient investigation of other neurological pathologies were excluded. By including cases from a variety of procedures, the findings of this study are more widely applicable to a range of specialties and clinicians. Furthermore, there is a widespread of demographics represented in the cohort of included patients, with cases originating from 27 countries across Asia, Europe, North America, and the Middle East.

### Study limitations

This study also has several limitations. A major limitation was the variable quality in reporting of cases. According to our quality assessment, it was deemed that only 57.6% of articles presented sufficient clinical information to replicate practice. As a result of the variability in reporting, the intricacies of individual management options, such as dosage, were not able to be accurately characterised or examined. Furthermore, the vast majority of articles were case reports without a specific focus on treatment, it is possible that certain administered treatments were not reported, such as intravenous fluids. Moreover, comparison between treatment options and patient discharge outcomes showed no statistically significant effect. This may be partially due to the small sample size of this study, but may also be attributable to the heterogeneity in the reporting of patient outcomes. Another limitation of note was that non-English articles were excluded from this study, which may have provided insightful data.

### Gaps in knowledge

This systematic review focussing on clinical management has brought to light several important deficiencies in the current understanding of CIN. Although we were able to identify the most commonly reported treatments utilised, the effectiveness and ultimate impact on patient prognosis and recovery has not been established. Furthermore, this investigation was not able to elucidate appropriate dosing and timing of reported treatments. Additionally, improved understanding of the pathophysiology of CIN will aid in the formulation of optimal clinical management strategies. Ultimately, this review only presents the currently utilised treatments for CIN from within the literature, and may not represent optimal management.

### Future directions

This study highlights the significant need for further investigation into the treatment of CIN. The overwhelming majority of the literature is formed by case reports and case series, which are low-level evidence, are highly biased, and are very difficult to draw practical conclusions from. Large cohort studies of patients who develop CIN following contrast-requiring procedures are required. In particular, studies should focus on specific treatment strategies and their effects on the short-term recovery, as well as the long-term outcomes of patients. Additional prognostic factors, including procedural details, comorbidities and risk factors, and contrast characteristics should also be methodically examined. Further investigation into the pathophysiology of CIN would not only improve our understanding of it as a clinical entity, but would provide an underpinning to treatment decision making.

## Conclusion

The findings of this review suggest that the clinical management of CIN could include aggressive intravenous fluids to reduce cerebrovascular exposure to contrast, corticosteroids to decrease inflammation and BBB disruption, ASMs to control seizure activity, and mannitol to reduce cerebral oedema. Nonetheless, we recommend each patient be treated on a case-by-case basis. Ultimately, the efficacy of different treatment options remain unclear, and larger cohort studies with a specific focus on management are required to define optimal treatment strategies.

### Supplementary Information

Below is the link to the electronic supplementary material.

Supplementary file1 (DOCX 44 KB)

## Data Availability

Not applicable.
