# Supplementary material for: Clinical management of contrast-induced neurotoxicity: a systematic review
Source: Acta Neurol Belg. 2024 Feb 8;124(4):1141–9. doi: 10.1007/s13760-024-02474-4 (PMC11266203; doi:10.1007/s13760-024-02474-4)
Supplement: Supplementary file 1 — Supplementary file1 (DOCX 44 KB) [file 13760_2024_2474_MOESM1_ESM.docx]

**Clinical Management of Contrast-induced Neurotoxicity: A Systematic Review**

Included Articlces^1-59^

1. Liao MT, Lin TT, Lin LY, Hwang JJ, Tseng CD. Contrast-induced encephalopathy after percutaneous coronary intervention. Acta Cardiologica Sinica. 2013;29(3):277-80.

2. Baguma M, Younan N, London F, Ossemann M, Vandermeeren Y. Contrast-associated transient cortical blindness: three cases with MRI and electrophysiology findings. Acta Neurologica Belgica. 2017;117(1):195-9.

3. Zhang F, Du X, Liu K. Contrast-induced encephalopathy following bronchial arteriography and endovascular procedure. Acta Neurologica Belgica. 2022.

4. Okuyan E, Biter HI, Yildiz SS, Dinckal MH. Transient cortical blindness following coronary angiography: A rare complication. American Journal of Case Reports. 2010;11:205-7.

5. Neilan PT, Urbine D. Contrast induced encephalopathy mimicking subarachnoid hemorrhage. American Journal of Respiratory and Critical Care Medicine Conference. 2019;199(9).

6. Kamata J, Fukami K, Yoshida H, Mizunuma Y, Moriai N, Takino T, et al. Transient cortical blindness following bypass graft angiography: a case report. Angiology. 1995;46(10):937-46.

7. Suri V, Agarwal R, Jadhao N, Ahuja GK. Cortical blindness after contrast-enhanced CT scan in a patient of sarcoidosis-Is it related to posterior reversible encephalopathy syndrome. Annals of Indian Academy of Neurology. 2011;14(4):298-300.

8. Nakao K, Joshi G, Hirose Y, Tanaka R, Yamada Y, Miyatini K, et al. Rare cases of contrast-induced encephalopathies. Asian Journal of Neurosurgery. 2020;15(03):786-93.

9. Li J, Qi G, Zhang H, Chen G, Wang S, Yan M, et al. Contrast-induced encephalopathy mimicking stroke after a second cerebral DSA: an unusual case report. BMC Neurology. 2021;21(1) (no pagination).

10. Babalova L, Ruzinak R, Ballova J, Sivak S, Kantorova E, Kurca E, et al. Contrast-induced encephalopathy. Bratislavske Lekarske Listy. 2021;122(9):618-20.

11. Xu SY, Song MM, Liu DY, Li CX, Xue LX, Li Y. Contrast-induced encephalopathy with significantly elevated levels of cerebrospinal fluid protein. British Journal of Neurosurgery. 2021.

12. Furlanis G, Manganotti P, Ajcevic M, Rossi J, Vincenzi C, Naccarato M, et al. CT Perfusion and EEG Patterns in Contrast-Induced Encephalopathy Stroke Mimic. Canadian Journal of Neurological Sciences. 2022;49(1):140-3.

13. Dattani A, Au L, Tay KH, Davey P. Contrast-Induced Encephalopathy following Coronary Angiography with No Radiological Features: A Case Report and Literature Review. Cardiology (Switzerland). 2018;139(3):197-201.

14. Guimaraens L, Vivas E, Fonnegra A, Sola T, Soler L, Balaguer E, et al. Transient encephalopathy from angiographic contrast: A rare complication in neurointerventional procedures. CardioVascular and Interventional Radiology. 2010;33(2):383-8.

15. Donepudi B, Trottier S. A Seizure and Hemiplegia following Contrast Exposure: Understanding Contrast-Induced Encephalopathy. Case Reports in Medicine. 2018;2018 (no pagination).

16. Allison C, Sharma V, Park J, Schirmer CM, Zand R. Contrast-Induced Encephalopathy after Cerebral Angiogram: A Case Series and Review of Literature. Case Reports in Neurology. 2021;13(2):405-13.

17. Rashid H, Brown J, Nix E, Fisher Covin A. Contrast-Induced encephalopathy following diagnostic coronary angiography. Clinical Case Reports. 2022;10(3) (no pagination).

18. Liu MR, Jiang H, Li XL, Yang P. Case report and literature review on low-osmolar, non-ionic iodine-based contrast-induced encephalopathy. Clinical Interventions In Aging. 2020;15:2277-89.

19. Simsek EC, Erturk E, Ucar R, Yilmaz AO, Ekmekci C, Mutlu I, et al. Transient Contrast Neurotoxicity After Percutaneous Coronary Intervention Mimicking Subarachnoid Hemorrhage in a Patient With Chronic Kidney Disease. Clinical Medicine Insights: Case Reports. 2019;12(no pagination).

20. Deb-Chatterji M, Schafer L, Grzyska U, Gelderblom M. Stroke-mimics: An acute brainstem syndrome after intravenous contrast medium application as a rare cause of contrast-induced neurotoxicity. Clinical Neurology and Neurosurgery. 2018;174:244-6.

21. Almasood A, Alsenani A, Alawlah A, Alsenani A. Contrast-Induced Encephalopathy Presenting With Fever After Coronary Angiography: A Case Report. Cureus. 2022;14(12).

22. Lee Z-V, Singh RSA. Transient cortical blindness after coronary angiography, bypass graft angiography, and coronary angioplasty. Cureus. 2021;13(1).

23. Saha A, Mitra S. Contrast-induced encephalopathy: a clinical conundrum. Cureus. 2022;14(11).

24. Oktaviono YH, Kawilarang MV, Kawilarang M, Gunadi RI, Philothra PT, Al Farabi MJ. Case Report: Transient cortical blindness following coronary angiography. F1000Research. 2022;10 (no pagination).

25. Hamra M, Bakhit Y, Khan M, Moore R. Case report and literature review on contrast-induced encephalopathy. Future Cardiology. 2017;13(4):331-5.

26. Spina R, Simon N, Markus R, Muller DWM, Kathir K. Recurrent contrast-induced encephalopathy following coronary angiography. Internal Medicine Journal. 2017;47(2):221-4.

27. Senoz O, Emren S, Ersecgin A, Emren Z, Bozkaya Y. Contrast-induced encephalopathy after primary percutaneous coronary angioplasty mimicking a subarachnoid hemorrhage. International Journal of the Cardiovascular Academy. 2020;6(4):183-5.

28. Leong S, Fanning NF. Persistent neurological deficit from iodinated contrast encephalopathy following intracranial aneurysm coiling: A case report and review of the literature. Interventional Neuroradiology. 2012;18(1):33-41.

29. Fernandes SI, Carvalho RJ, Santos LM, Sá FM, Antunes JA, Mendes AS, et al. Transient cortical blindness following coronary angiography. JACC: Case Reports. 2019;1(2):188-91.

30. Borghi C, Saia F, Marzocchi A, Branzi A. The conundrum of transient cortical blindness following coronary angiography. Journal of Cardiovascular Medicine. 2008;9(10):1063-5.

31. Park JC, Ahn JH, Chang IB, Oh JK, Kim JH, Song JH. A case of unusual presentation of contrast-induced encephalopathy after cerebral angiography using iodixanol. Journal of cerebrovascular and endovascular neurosurgery. 2017;19(3):184-8.

32. Haussen DC, Modir R, Yavagal DR. Unilateral contrast neurotoxicity as a stroke mimic after cerebral angiogram. Journal of Neuroimaging. 2013;23(2):231-3.

33. Eleftheriou A, Rashid AS, Lundin F. Late Transient Contrast-Induced Encephalopathy after Percutaneous Coronary Intervention. Journal of Stroke and Cerebrovascular Diseases. 2018;27(6):e104-e6.

34. Akhtar N, Khatri IA, Naseer A, Ikram J, Ahmed W. Transient cortical blindness after coronary angiography: A case report and literature review. Journal of the Pakistan Medical Association. 2011;61(3):295-7.

35. Vallabhaneni R, Jim J, Derdeyn CP, Sanchez LA. Transient cortical blindness after thoracic endovascular aneurysm repair. Journal of vascular surgery. 2011;53(5):1405-8.

36. Aykan A, Zehir R, Karabay CY, Kocabay G. Contrast− induced monoplegia following coronary angioplasty with iopromide. Kardiologia Polska (Polish Heart Journal). 2012;70(5):499-500.

37. Lim KK, Radford DJ. Transient cortical blindness related to coronary angiography and graft study. The Medical Journal of Australia. 2002;177(1):43-4.

38. Qiu T, Dai X, Gong Q, Pu R, Xiao H, Shi Q, et al. Transient oculomotor paralysis after cerebral angiography: A case report. Medicine. 2021;100(22).

39. Lo LW, Chan HF, Ma KF, Cheng LF, Chan TK. Transient cortical blindness following vertebral angiography: a case report. Neurointervention. 2015;10(1):39-42.

40. Montejo C, Rodriguez A, Pascual-Vicente M, Renu A. Transient contrast-induced encephalopathy after internal carotid artery embolisation prior to surgery for nasopharyngeal carcinoma. Neurologia. 2020;35(4):287-9.

41. Matsubara N, Izumi T, Miyachi S, Ota K, Wakabayashi T. Contrast-induced encephalopathy following embolization of intracranial aneurysms in hemodialysis patients. Neurologia Medico-Chirurgica. 2017;57(12):641-8.

42. Shinoda J, Ajimi Y, Yamada M, Onozuka S. Cortical Blindness During Coil Embolization of an Unruptured Intracranial Aneurysm—Case Report—. Neurologia medico-chirurgica. 2004;44(8):416-9.

43. Vigano’ M, Mantero V, Basilico P, Cordano C, Sangalli D, Reganati P, et al. Contrast-induced encephalopathy mimicking total anterior circulation stroke: a case report and review of the literature. Neurological Sciences. 2021;42:1145-50.

44. Sharp S, Stone J, Beach R. Contrast agent neurotoxicity presenting as subarachnoid hemorrhage. Neurology. 1999;52(7):1503-5.

45. Monforte M, Marca GD, Lozupone E. Contrast-induced Encephalopathy. Neurology India. 2020;68(3):718-9.

46. Riahi L, Mediouni M, Messelmani M, Fehri W. A Singular Manifestation of Contrast-induced Encephalopathy Following Coronary Angiography. Neurology India. 2019;67(6):1525-7.

47. Sadiq MA, Al Habsi MS, Nadar SK, Shaikh MM, Baomar HA. Transient contrast induced neurotoxicity after coronary angiography: A contrast re-challenge case. Pakistan Journal of Medical Sciences. 2020;36(5):1140-2.

48. Kocabay G, Karabay CY. Iopromide-induced encephalopathy following coronary angioplasty. Perfusion. 2011;26(1):67-70.

49. Ozelsancak R, Erken E, Yildiz I, Giray S, Yildirim T, Micozkadioglu H. A very rare case of encephalopathy in a patient with end-stage renal disease: Contrast agent, ioversol. Renal Failure. 2010;32(9):1128-30.

50. Yan J, Ramanathan V. Severe Encephalopathy Following Cerebral Arteriogram in a Patient with End-Stage Renal Disease. Seminars in Dialysis. 2013;26(2):203-7.

51. Kwok B, Lim T. Cortical blindness following coronary angiography. Singapore medical journal. 2000;41(12):604-5.

52. Sawaya RA, Hammoud R, Arnaout S, Alam S. Contrast-induced encephalopathy following coronary angioplasty with iohexol. Southern Medical Journal. 2007;100(10):1054-5.

53. Spiriev T, Laleva L, Alioski N, Dobrikov R, Gelev V, Milev M, et al. Contrast-induced neurotoxicity presented as transient cortical blindness after stent-assisted coiling of a medium-sized unruptured basilar artery aneurysm: A case report and review of the literature. Surgical Neurology International. 2022;13 (no pagination).

54. Yazici M, Ozhan H, Kinay O, Kilicaslan B, Karaca M, Cece H, et al. Transient cortical blindness after cardiac catheterization with iobitridol. Texas Heart Institute Journal. 2007;34(3):373-5.

55. Kahyaolu M, Aca M, Cakmak EO, Gecmen C, Zgi BA. Contrast-induced encephalopathy after percutaneous peripheral intervention. Turk Kardiyoloji Dernegi Arsivi. 2018;46(2):140-2.

56. Menna D, Capoccia L, Rizzo A, Sbarigia E, Speziale F. An atypical case of contrast-induced encephalopathy after carotid artery stenting. Vascular. 2013;21(2):109-12.

57. Helsley JD. Cortical blindness following cerebral angiography. The West Virginia medical journal. 1995;91(7):324.

58. Tong X, Hu P, Hong T, Li M, Zhang P, Li G, et al. Transient Cortical Blindness Associated with Endovascular Procedures for Intracranial Aneurysms. World Neurosurgery. 2018;119:123-31.

59. Zevallos CB, Dandapat S, Ansari S, Farooqui M, Quispe-Orozco D, Mendez-Ruiz A, et al. Clinical and Imaging Features of Contrast-Induced Neurotoxicity After Neurointerventional Surgery. World Neurosurgery. 2020;142:e316-e24.
